# Ventilatory efficiency is superior to peak oxygen uptake for prediction of lung resection cardiovascular complications

**DOI:** 10.1371/journal.pone.0272984

**Published:** 2022-08-12

**Authors:** Andrej Mazur, Kristian Brat, Pavel Homolka, Zdenek Merta, Michal Svoboda, Monika Bratova, Vladimir Sramek, Lyle J. Olson, Ivan Cundrle

**Affiliations:** 1 Department of Anesthesiology and Intensive Care, St. Anne’s University Hospital Brno, Brno, Czech Republic; 2 Faculty of Medicine, Masaryk University, Brno, Czech Republic; 3 International Clinical Research Center, St. Anne’s University Hospital Brno, Brno, Czech Republic; 4 Department of Respiratory Diseases, University Hospital Brno, Czech Republic; 5 Department of Sports Medicine and Rehabilitation, St. Anne’s University Hospital, Brno; 6 Institute of Biostatistics and Analyses, Ltd., Brno, Czech Republic; 7 Department of Cardiovascular Diseases, Mayo Clinic, Rochester, MN, United States of America; University of Brasilia: Universidade de Brasilia, BRAZIL

## Abstract

**Introduction:**

Ventilatory efficiency (V_E_/VCO_2_ slope) has been shown superior to peak oxygen consumption (VO_2_) for prediction of post-operative pulmonary complications in patients undergoing thoracotomy. V_E_/VCO_2_ slope is determined by ventilatory drive and ventilation/perfusion mismatch whereas VO_2_ is related to cardiac output and arteriovenous oxygen difference. We hypothesized pre-operative VO_2_ predicts post-operative cardiovascular complications in patients undergoing lung resection.

**Methods:**

Lung resection candidates from a published study were evaluated by post-hoc analysis. All of the patients underwent preoperative cardiopulmonary exercise testing. Post-operative cardiovascular complications were assessed during the first 30 post-operative days or hospital stay. One-way analysis of variance or the Kruskal–Wallis test, and multivariate logistic regression were used for statistical analysis and data summarized as median (IQR).

**Results:**

Of 353 subjects, 30 (9%) developed pulmonary complications only (excluded from further analysis), while 78 subjects (22%) developed cardiovascular complications and were divided into two groups for analysis: cardiovascular only (n = 49) and cardiovascular with pulmonary complications (n = 29). Compared to patients without complications (n = 245), peak VO_2_ was significantly lower in the cardiovascular with pulmonary complications group [19.9 ml/kg/min (16.5–25) vs. 16.3 ml/kg/min (15–20.3); *P*<0.01] but not in the cardiovascular only complications group [19.9 ml/kg/min (16.5–25) vs 19.0 ml/kg/min (16–23.1); *P* = 0.18]. In contrast, V_E_/VCO_2_ slope was significantly higher in both cardiovascular only [29 (25–33) vs. 31 (27–37); *P* = 0.05] and cardiovascular with pulmonary complication groups [29 (25–33) vs. 37 (34–42); *P*<0.01)]. Logistic regression analysis showed V_E_/VCO_2_ slope [OR = 1.06; 95%CI (1.01–1.11); *P* = 0.01; AUC = 0.74], but not peak VO_2_ to be independently associated with post-operative cardiovascular complications.

**Conclusion:**

V_E_/VCO_2_ slope is superior to peak VO_2_ for prediction of post-operative cardiovascular complications in lung resection candidates.

## Introduction

Peak oxygen consumption (VO_2_) is recommended by both American and European guidelines for pre-operative risk stratification of lung resection candidates [1,2]. However, over the last decade, several studies have shown peak VO_2_ to be a poor predictor of post-operative pulmonary complications [3] and inferior to ventilatory efficiency (V_E_/VCO_2_ slope) [4,5].

By the alveolar gas equation ventilatory efficiency is defined as V_E_/VCO_2_ = 863 / (PaCO_2_ x (1—V_D_ /V_T_)) [6]. Therefore, V_E_/VCO_2_ is increased by lower PaCO_2_ (hyperventilation) or increase of the V_D_ /V_T_ ratio (ventilation/perfusion mismatch or ineffective breathing pattern with rapid and shallow breathing) which may explain its superior prediction of post-operative pulmonary complications [6].

In contrast, determinants of VO_2_ include cardiac output [7] and arteriovenous oxygen difference. Peak VO_2_ is considered a cardiovascular fitness parameter [8] and was shown to be a major risk factor for post-operative cardiovascular complications [9]. Hence, an association with cardiovascular complications might be expected.

We hypothesized peak exercise VO_2_ predicts post-operative cardiovascular complications in patients undergoing thoracic surgery. Accordingly, the aim of this study was to compare pre-operative peak exercise VO_2_ and V_E_/VCO_2_ slope in the prediction of post-operative cardiovascular complications in patients after lung resection surgery.

## Methods

### Subject selection

Subjects included were consecutive patients who underwent lung resection surgery. Inclusion criteria were age ≥18 years and ability to undergo cardiopulmonary exercise testing (CPET). Exclusion criteria were contraindication for lung resection because of inoperability or low peak predicted post-operative VO_2_ [VO_2_<10ml/kg/min or <35% predicted together with predicted post-operative forced expiratory volume at one second (FEV_1_) <30% and diffusing capacity for carbon monoxide (DL_CO_) <30% ^2^)] and development of pulmonary complications only.

### Study design

This is a post-hoc analysis of a previously published prospective multicenter study which evaluated pre-operative rest ventilatory parameters as predictors of post-operative pulmonary complications in lung resection candidates [10]. All of the included patients underwent preoperative CPET and pulmonary function tests. Post-operative cardiovascular complications were prospectively accessed from the first 30 post-operative days or from the hospital stay. The study was conducted in accordance with the declaration of Helsinki. All participants provided written informed consent and the study was approved by the local Ethics Committee of St. Anne’s University Hospital in Brno (reference No. 19JS/2017, date of approval April 12, 2017; reference No. 2G/2018, date of approval March 21^st^, 2018) and by the local Ethics Committee of the University Hospital Brno (reference No. 150617/EK, date of approval June 19^th^, 2017). The study was registered at ClinicalTrials.gov (NCT03498352) and the manuscript adheres to the applicable STROBE guidelines.

### Cardiopulmonary exercise testing

Exercise capacity was assessed by CPET as described previously [10]. Subjects underwent preoperative CPET (symptom-limited) using a bicycle ergometer (Ergoline, Ergometrics 800^®^, Bitz, Germany). Expired gas analysis was done with the use of a PowerCube-Ergo^®^ cardiopulmonary exercise system (Ganshorn Medizin Electronic GmbH, Niederlauer, Germany). Arterial blood gas analysis was done twice (at rest and peak exercise) using the ABL90 Flex Plus^®^ (Radiometer Medical ApS, Denmark) device. The CPET protocol included a warm-up phase without resistance (60 seconds) followed by a linearly increasing (15 W/min) ramp protocol. Measured parameters included VO_2_, carbon dioxide output (VCO_2_), breathing frequency (f_b_), tidal volume (V_T_), minute ventilation (V_E_) and P_ET_CO_2_. The derived CPET parameters included dead space to tidal volume ratio (V_D_/V_T_), respiratory exchange ratio (RER) and V_E_/VCO_2_ slope.

### Pulmonary function tests

Spirometry was performed using the ZAN100 device (nSpire Health, Inc., USA) and DL_CO_ using the PowerCube Diffusion+ device (Ganshorn Medizin Electronic GmbH, Germany). Analyzed parameters include forced vital capacity (FVC), forced expiratory volume in one second (FEV_1_) and FEV_1_/FVC ratio and were reported as percent (%) of predicted value.

### Cardiovascular complications

Cardiovascular complications were defined as previously recommended [11] and prospectively assessed from the first 30 post-operative days or from the hospital stay including arrhythmias (atrial fibrillation, supraventricular tachycardia–duration ≥ 10 seconds [12]); hypotension (catecholamine administration or parenteral fluids >200ml/h); heart failure (recently elevated serum brain natriuretic peptide and/or administration of inotropes); pulmonary edema (chest X-ray + clinical signs); pulmonary embolism (CT-angiography or highly suspicious right heart echocardiography signs); myocardial infarction/minimal myocardial lesion (coronary angiography or new ECG signs with serum positive troponin), stroke (newly developed neurological deficit with CT signs of brain infarction of ischemic or hemorrhagic etiology [13]) and cardiopulmonary resuscitation.

### Pulmonary complications

Pulmonary complications were assessed as previously described by our group [10] and others [4,14] and included pneumonia; atelectasis; respiratory failure requiring mechanical ventilation; adult respiratory distress syndrome and tracheostomy. ICU length of stay (LOS), hospital LOS and 30-day mortality were also monitored. Surgical Mortality Probability Model (S-MPM), an ASA (American Society of Anesthesiologists) physical status adjusted for surgery risk class and emergency status [15], was used to allow patient’s co-morbidities comparison.

### Statistics

The Shapiro-Wilk test was used to evaluate normality. One-way analysis of variance or the Kruskal–Wallis test by ranks followed by either post-hoc Tukey HSD test or Mann–Whitney U test (as appropriate) were used to test for differences among the groups. Chi-square test was used to compare categorical variables. Univariate logistic regression was done for parameters significantly different between groups. The Spearman rank test was performed to evaluate parameters with significant correlation. Parameters with correlation coefficients <±0.5 were used for multivariate stepwise logistic and linear regression. A multivariate stepwise logistic regression model was constructed to analyze which parameters were independently associated with the development of cardiovascular complications, ICU readmission and 30-day mortality. To evaluate model performance receiver operating characteristic (ROC) analyses were performed. Multivariate stepwise linear regression analyses (using same parameters as for multivariate logistic regression) were performed to evaluate the association of peak VO_2_ and V_E_/VCO_2_ slope with hospital and ICU LOS. Data are summarized as mean ± SD or median (IQR) as appropriate; *P* values <0.05 were considered statistically significant. Statistica software 12.0 (StatSoft Inc., Prague, Czech Republic) and IBM SPSS Statistics 25.0 (IBM Corp, USA) were used for analysis.

## Results

Data from 353 lung resection surgery candidates from our previous study [10] were analyzed. Thirty patients (9%) developed pulmonary complications only and were excluded; 78 patients (22%) developed cardiovascular complications and 245 patients (69%) had no complications. Comparison of patients with and without cardiovascular complications is shown in S1 Table. Out of the 78 patients with cardiovascular complications, 49 patients developed cardiovascular complications only and 29 patients developed cardiovascular with pulmonary complications. Cardiovascular and pulmonary complications often coexist [16], and despite prospective assessment of complications, we were not able to fully distinguish which complications were primary and which were secondary. To mitigate this, we divided our cardiovascular complications subjects into 2 groups–cardiovascular only and cardiovascular with pulmonary complications (frequency of cardiovascular complications was nearly the same in both groups Table 1) and analyzed them separately.

**Table 1:** Cardiovascular complications: Type and frequency (n = 78).

	Cardiovascular only (n = 49)	With pulmonary (n = 29)	*P*
Lung edema n (%)	0	2 (7)	0.14
Pulmonary embolism n (%)	3 (6)	1 (3)	1.00
Arrhythmia n (%)	26 (53)	21 (72)	0.10
Hypotension n (%)	26 (53)	19 (66)	0.35
Heart failure n (%)	0	2 (7)	0.14
Acute myocardial infarction n (%)	0	0	1.00
Cardiopulmonary resuscitation n (%)	0	3 (10)	0.05
Ischemic stroke n (%)	0	2 (7)	0.14

Subject and surgery characteristics are shown in Table 2. Subjects in both the post-operative cardiovascular complications groups were significantly older and had higher S-MPM and comorbidities. Hospital and ICU LOS was significantly longer and frequency of ICU readmissions higher in both cardiovascular complication groups. The 30-day mortality was significantly higher in the cardiovascular with pulmonary complications group.

**Table 2:** Subject characteristics and surgery comparison.

parameter	Without complications (n = 245)	Cardiovascular complications (n = 78)			ANOVA *P*
		Cardiovascular only (n = 49)	With pulmonary (n = 29)		
Male n (%)	135 (55)	26 (53)	20 (69)		0.33
Age (years)	65 (55–70)	67 (63–74)*	70 (65–73)**		<0.01
BMI (kg/m^2^)	28.2 (24.4–31.5)	27.4 (23.8–29.9)	29.1 (25.3–32)		0.64
S-MPM	4 (4–6)	6 (4–6)**	6 (6–6)**		<0.01
Hypertension n (%)	114 (47)	32 (65)*	24 (83)**		<0.01
Ischemic heart disease n (%)	17 (7)	7 (14)	5 (17)		0.07
COPD/asthma n (%)	37 (15)	7 (14)§§	15 (52)**		<0.01
Diabetes mellitus n (%)	31 (13)	10 (20)	8 (28)		0.06
Stroke n (%)	10 (4)	6 (12)*	5 (17)*		<0.01
**Surgery**					
Thoracotomy n (%)	123 (50)	28 (57)		22 (76)**	0.03
Lobectomy n (%)	106 (43)	29 (64)*§		24 (83)**	<0.01
Bilobectomy n (%)	6 (15)	3 (1)		0	0.23
Pneumonectomy n (%)	5 (2)	5 (10)**		1 (3)	0.02
Wedge resection n (%)	128 (52)	12 (24)**		4 (14)**	<0.01
**Post-operative Outcome**					
Hospital LOS (days)	6 (5–8)	8 (7–12)**§§		16 (12–26)**	<0.01
ICU LOS (days)	3 (2–4)	5 (4–7)**§§		10 (8–13)**	<0.01
ICU readmission n (%)	1 (0.4)	2 (4)*§§		8 (28)**	<0.01
30-day mortality n (%)	2 (1)	0§§		5 (17)**	<0.01

* = *P*<0.05

** = *P*<0.01 vs without complications group.

§ = *P*<0.05

§§ = *P*<0.01 vs cardiovascular with pulmonary group.

BMI = body mass index; COPD = Chronic obstructive pulmonary disease; ICU = intensive care unit; LOS = length of stay; PPC = post-operative pulmonary complications; S-MPM = Surgical Mortality Probability Model.

Results of pulmonary function tests and arterial blood gases are summarized in Table 3. FEV_1_%, FVC%, FEV_1_/FVC% and DL_CO_% and rest PaO_2_ were significantly lower in the cardiovascular with pulmonary complications group. FEV_1_/FVC% was also lower in the cardiovascular complications only group.

**Table 3:** Pulmonary function tests and arterial blood gases comparison.

parameter	Without complications (n = 245)		Cardiovascular complications (n = 78)		ANOVA *P*
			Cardiovascular only (n = 49)	With pulmonary (n = 29)	
**Pulmonary Function Tests**					
FEV_1_ (%predicted)		94 ± 18	87 ± 19	82 ± 20**	<0.01
FVC (%predicted)		95 ± 16	92 ± 18	87 ± 18*	0.02
FEV_1_/FVC (%)		82 (76–87)	79 (70–85)*	77 (68–84)*	0.01
DL_CO_ (%predicted)		84 ± 21	79 ± 26	71 ± 23**	0.01
**Rest Arterial Blood Gases**					
PaO_2_ (mmHg)		79 ± 9	76 ± 8	74 ± 7*	0.01
PaCO_2_ (mmHg)		36 (33–38)	35 (33–38)	35 (32–36)	0.12
pH		7.45 (7.43–7.46)	7.44 (7.43–7.46)	7.44 (7.42–7.46)	0.45
**Peak Exercise Arterial Blood Gases**					
PaO_2_ (mmHg)		86 (79–92)	86 (77–92)	78 (74–89)	0.13
PaCO_2_ (mmHg)		36 ± 5	35 ± 4	35 ± 5	0.23
pH		7.37 (7.33–7.39)	7.36 (7.34–7.39)	7.37 (7.33–7.41)	0.49

* = *P*<0.05

** = *P*<0.01 vs without complications group.

§ = *P*<0.05

§§ = *P*<0.01 vs cardiovascular with pulmonary group.

DL_CO_ = diffusing lung capacity for carbon monoxide; FEV_1_ = forced expiratory volume in one second; FVC = forced vital capacity; PaCO_2_ = arterial partial pressure of carbon dioxide; PaO_2_ = arterial partial pressure of oxygen.

Rest and exercise ventilatory parameters for all groups are summarized in Table 4. At rest, P_ET_CO_2_ was significantly lower in both cardiovascular complication groups. At peak exercise, VO_2_, VCO_2_, RER and V_T_ were significantly lower and f_b_ significantly higher in the cardiovascular with pulmonary complications group. Peak exercise P_ET_CO_2_ was lower and V_E_/VCO_2_ slope higher in both cardiovascular complication groups.

**Table 4:** Cardiopulmonary exercise testing comparison.

parameter	Without complications (n = 245)	Cardiovascular complications (n = 78)		ANOVA *P*
		Cardiovascular only only (n = 49)	With pulmonary (n = 29)	
**Rest Ventilation and Gas Exchange**				
VO_2_ (ml/kg/min)	4.2 (3.5–5.0)	4.2 (3.1–4.6)	4.1 (3.4–5.2)	0.51
VCO_2_ (ml/min)	0.25 ± 0.10	0.22 ± 0.08	0.22 ± 0.10	0.04
V_E_ (l/min)	10 ± 4	9 ± 3	10 ± 5	0.47
V_T_ (ml)	0.57 (0.40–0.74)	0.57 (0.43–0.71)	0.45 (0.31–0.65)	0.19
f_b_ (bpm)	18 (15–21)	17 (14–21)§§	20 (18–23)*	0.01
V_D_/V_T_	0.29 (0.23–0.34)	0.30 (0.24–0.34)	0.29 (0.23–0.34)	0.77
P_ET_CO_2_ (mmHg)	30 (27–32)	28 (24–31)*§	26 (23–28)**	<0.01
**Peak Exercise Ventilation and Gas Exchange**				
VO_2_ (ml/kg/min)	19.9 (16.5–25)	19.0 (16–23.1)	16.3 (15–20.3)**	0.01
VCO_2_ (ml/min)	1.67 (1.30–2.10)	1.56 (1.23–1.95)	1.32 (1.03–1.71)**	<0.01
RER	1.05 (0.92–1.14)	0.99 (0.88–1.12)	0.97 (0.82–1.05)**	0.01
V_E_ (l/min)	54 (44–68)	53 (41–65)	48 (43–61)	0.31
V_T_ (ml)	1.75 (1.37–2.11)	1.74 (1.27–2.14)	1.42 (1.08–1.85)**	0.01
f_b_ (bpm)	32 (28–36)	33 (30–37)	35 (32–41)**	0.01
V_D_/V_T_	0.21 ± 0.07	0.22 ± 0.06	0.24 ± 0.06	0.12
P_ET_CO_2_ (mmHg)	36 ± 5	33 ± 6*§	30 ± 5**	<0.01
V_E_/VCO_2_ slope	29 (25–33)	31 (27–37)*§§	37 (34–42)**	<0.01

* = *P*<0.05

** = *P*<0.01 vs without complications group.

§ = *P*<0.05

§§ = *P*<0.01 vs cardiovascular with pulmonary group.

f_b_ = breathing frequency; P_ET_CO_2_ = partial pressure of end-tidal carbon dioxide; PPC = post-operative pulmonary complications; RER = respiratory exchange ratio; VCO_2_ = carbon dioxide output; V_D_ = dead space volume; V_E_ = minute ventilation; V_E_/VCO_2_ = ventilatory efficiency; VO_2_ = oxygen consumption; V_T_ = tidal volume.

Univariate logistic regression demonstrated V_E_/VCO_2_ slope to be significantly associated with cardiovascular complications (OR = 1.10; 95%CI 1.06–1.15; *P*<0.01; AUC = 0.67) and ICU readmissions (OR = 0.92; 95% CI 0.85–0.99; *P* = 0.02; AUC = 0.71). Patients with V_E_/VCO_2_ slope ≥34.8 had significantly higher probability (17.4% vs. 46.1%) for post-operative cardiovascular complications with OR 4.1 (95% CI 2.3–7.1; *P*<0.01). However, V_E_/VCO_2_ slope association with 30-day mortality was not significant (OR = 0.93; 95%CI 0.85–1.01; *P* = 0.08; AUC = 0.68). While peak VO_2_ was significantly associated with cardiovascular complications (OR = 0.93; 95% CI 0.89–0.98; *P*<0.01; AUC = 0.60), it was not associated with ICU readmissions (OR = 1.05; 95% CI 0.95–1.16; *P* = 0.36; AUC = 0.56) or 30-day mortality (OR = 1.11; 0.97–1.28; *P* = 0.14; AUC = 0.61).

**Fig 1:**
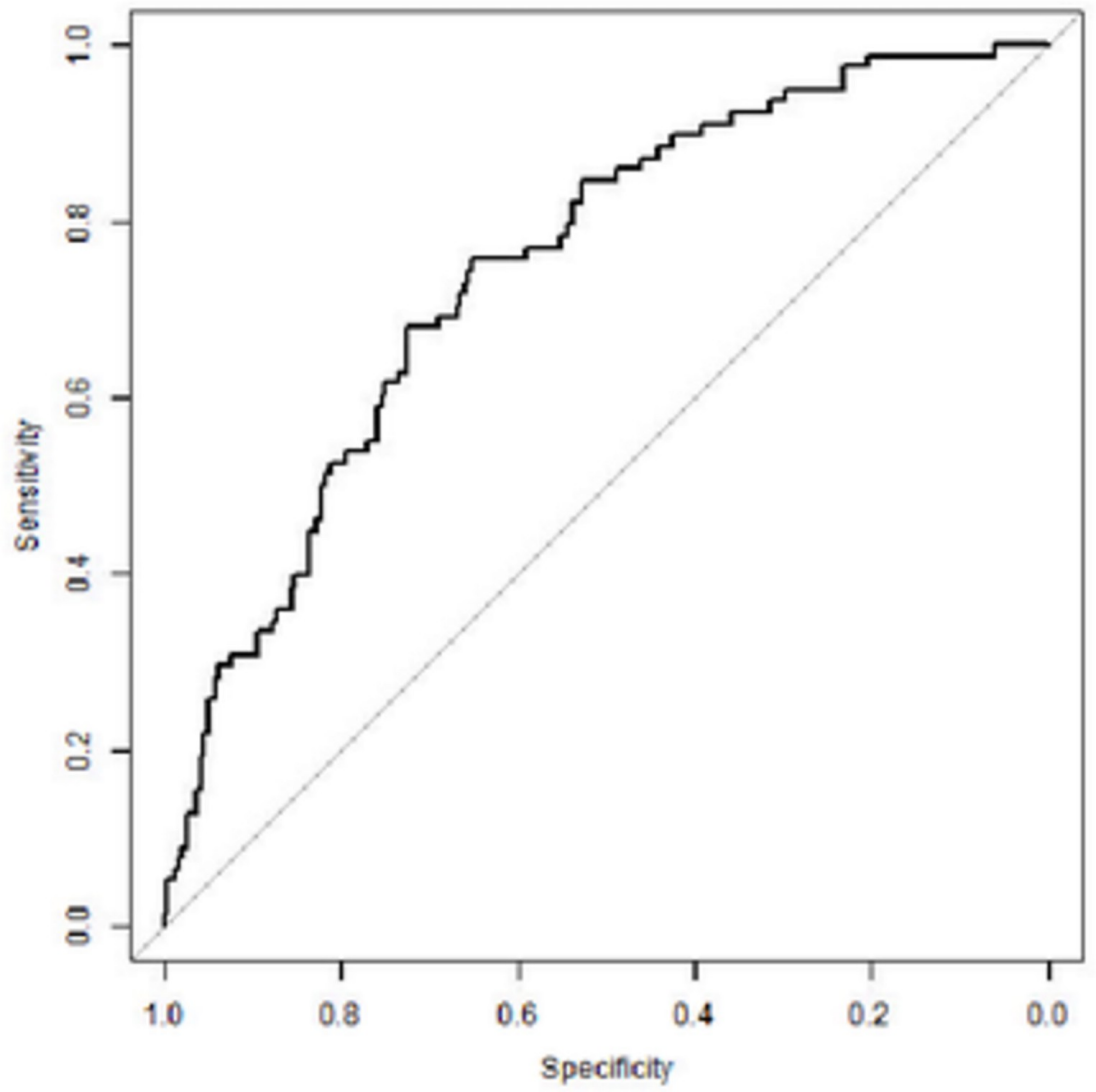
Receiver operating characteristic curve of V_E_/VCO_2_ slope and post-operative cardiovascular complications. Multivariate logistic regression model [AUC = 0.747 (0.687; 0.807)].

Multivariate logistic regression showed V_E_/VCO_2_ slope, age, and wedge resection, but not peak VO_2_ to be independently associated with post-operative cardiovascular complications (Table 5) with AUC 0.75 (Fig 1). V_E_/VCO_2_ slope also remained significantly associated with ICU readmissions (OR = 0.92; 95% CI 0.86–0.99; *P* = 0.03; AUC = 0.70).

**Table 5:** Logistic regression analysis.

	univariate			multivariate stepwise		
	OR	95 CI	p	OR	95 CI	*P*
Age	1.05	1.02–1.08	<0.01	1.04	1.01–1.07	0.02
S-MPM	1.63	1.29–2.07	<0.01	NS		
Thoracotomy No (%)	1.77	1.05–3.00	0.03	NS		
Lobectomy No (%)	2.78	1.62–4.76	<0.01	NS		
Wedge resection No (%)	0.24	0.13–0.43	<0.01	0.28	0.15–0.53	<0.01
FEV_1_/FVC	0.97	0.94–0.99	<0.01	NS		
DL_CO_	0.98	0.97–1.00	0.01	NS		
peak VO_2_	0.93	0.89–0.98	<0.01	NS		
V_E_/VCO_2_ slope	1.10	1.06–1.15	<0.01	1.06	1.01–1.11	0.01

DL_CO_ = diffusing lung capacity for carbon monoxide; FEV_1_ = forced expiratory volume in one second; FVC = forced vital capacity; P_ET_CO_2_ = partial pressure of end-tidal carbon dioxide; S-MPM = Surgical Mortality Probability Model; V_E_/VCO_2_ = ventilatory efficiency; VO_2_ = oxygen consumption.

Both peak VO_2_ and V_E_/VCO_2_ significantly correlated with ICU LOS (rho = -0.13, *P* = 0.02; rho = 0.38, *P*<0.01, respectively) and hospital LOS (rho = -0.13, *P* = 0.02; rho = 0.34, *P*<0.01, respectively). Multiple stepwise regression analysis showed V_E_/VCO_2_ slope, but not peak VO_2_ to be significantly associated with ICU LOS (b = 0.13, F = 9, *P*<0.01) and hospital LOS (b = 0.19, F9.7, *P*<0.01).

## Discussion

A major finding of this post-hoc analysis of a previously reported surgical cohort was that peak exercise VO_2_ did not predict post-operative cardiovascular complications, ICU readmissions, mortality, and hospital and ICU LOS in lung resection candidates. However, in contrast V_E_/VCO_2_ slope predicted post-operative cardiovascular complications, ICU readmissions, and hospital and ICU LOS.

The incidence of post-operative cardiovascular complications was 22%, similar to previous reports [17,18]. The most frequent cardiovascular complication was supraventricular arrhythmia (60%) in agreement with previous studies of post-lung resection surgery [18,19]. The second most common complication was post-operative hypotension (58%), which is also frequent [20]. Although these complications may be considered mild, post-operative arrhythmias are associated with longer hospital LOS, intensive care unit admissions, resource utilization and costs of care [17,21] and post-operative hypotension may be associated with myocardial injury [20]. Indeed, in our cohort, ICU readmissions were greater and hospital and ICU LOS were significantly longer in both cardiovascular complication groups. Severe complications (except of hypotension and arrhythmia) were relatively rare (16%) and developed in only 10 patients.

Compared to patients without post-operative complications, patients who developed cardiovascular complications (with or without pulmonary complications) were significantly older, had more comorbidities and lower pre-operative FEV_1_/FVC%. Lobectomy was more frequent and wedge resection less frequent in these patients, in concordance with previous studies [17,18]. In our study, patients with a combination of cardiovascular and pulmonary complications had previously established risk factors for post-operative pulmonary complications including thoracotomy, lower FEV_1_%, FVC%, DL_CO_% and PaO_2_ [22,23]. The 30-day mortality was also higher in this group of patients.

During pre-operative exercise, patients with post-operative cardiovascular complications (with or without pulmonary complications) exhibited significantly higher V_E_/VCO_2_ slope. In contrast, peak VO_2_ was significantly lower only in the cardiovascular with pulmonary complications group. V_E_/VCO_2_ slope is determined by ventilatory control impairment [6] suggesting it may be a parameter which is predictive of post-operative pulmonary complications. Indeed, several studies have previously shown its superiority to peak VO_2_ [4,5,10,14,24,25]. However, the main determinant of peak VO_2_ is cardiac output [7], which has been shown to be a major risk factor for post-operative cardiovascular complications [9].

Peak VO_2_ is considered a parameter for assessment of cardiovascular fitness [8]. Hence, an association with cardiovascular complications might be expected. However, in this study, although the peak VO_2_ was significantly associated with cardiovascular complications by univariate analysis, multivariate logistic regression showed that only V_E_/VCO_2_ slope, type of surgical procedure and age were independently associated with cardiovascular complications. This is in agreement with prior studies in chronic heart failure patients where V_E_/VCO_2_ slope has also been found superior to peak VO_2_ in the prediction of cardiovascular events [26–28]. The observed association of V_E_/VCO_2_ slope and cardiovascular complications might be explained by an inverse association of V_E_/VCO_2_ slope with cardiac output and positive association with capillary wedge pressure [29]. Moreover, unlike peak VO_2_, V_E_/VCO_2_ slope is independent of patient effort and peripheral skeletal muscle function enabling acquisition during submaximal exercise (V_E_/VCO_2_ has been validated across different RER [30]) which may be beneficial for assessment of functionally limited lung surgery candidates.

Our findings may have clinical implications. First, peak VO_2_ is part of risk stratification algorithms in both American and European guidelines [1,2]. However, multiple studies have previously shown its poor prognostic utility [3–5,10]. In contrast, V_E_/VCO_2_ slope has been shown to be significantly better in the prediction of pulmonary [4,5,10,14,24,25] and now also cardiovascular complications. Accordingly, we suggest that lung resection candidate risk stratification algorithms consider routine inclusion of V_E_/VCO_2_ slope. Second, pre-operative risk stratification may allow pre-operative patient optimization as V_E_/VCO_2_ slope may be a therapeutic target [31] and promote better perioperative planning [17].

This study has several limitations. First, according to published ERS/ESTS guidelines [2], subjects with peak predicted post-operative VO_2_<10ml/kg/min were excluded, which may diminish the predictive value of peak VO_2_. However, only 6 subjects from our study cohort were excluded for this reason. Second, chronic medication data were unavailable. However, chronic medication is usually associated with the underlying diseases and our logistic regression analysis was adjusted for those. Third, PaO_2_ was significantly lower in patients with cardiovascular and pulmonary complications at rest. As VO_2_ is determined not only by cardiac output but also arteriovenous oxygen difference, this may have contributed to the observed VO_2_ differences (although these were observed only at peak exercise). Therefore, we cannot exclude, that peak VO_2_ may be a relevant prognostic factor in this subgroup of severely ill patients.

## Conclusion

Peak VO_2_ is a poor predictor of post-operative cardiovascular complications. In contrast, the V_E_/VCO_2_ slope predicted post-operative cardiovascular complications, ICU readmissions and both hospital and ICU LOS. Our observations suggest consideration of routine inclusion of V_E_/VCO_2_ slope for pre-operative risk stratification in lung resection candidates.

## Supporting information

S1 Table Comparison of patients with and without cardiovascular complications. Comparison of patients with and without cardiovascular complications.
(DOCX)

S2 Table Dataset. Minimal dataset.
(XLSX)
