# Supplementary material for: Ventilatory efficiency is superior to peak oxygen uptake for prediction of lung resection cardiovascular complications
Source: PLoS One. 2022 Aug 12;17(8):e0272984. doi: 10.1371/journal.pone.0272984 (PMC9374210; doi:10.1371/journal.pone.0272984)
Supplement: S1 Table — Comparison of patients with and without cardiovascular complications. (DOCX) [file pone.0272984.s001.docx]

| **Supplement Table 1. Comparison of patients with and without cardiovascular complications** | | | | |
| --- | --- | --- | --- | --- |
| Parameter | Without complications (n=245) | Cardiovascular complications (n=78) | | p |
|  |  |  |  |  |
| **Subject characteristics** | | | | |
| Male n(%) | 135 (55) | 46 (59) | 0.60 | |
| Age (years) | 65 (55-70) | 68 (63-74) | <0.01 | |
| BMI (kg/m^2^) | 28.2 (24.4-31.5) | 27.9 (24.6-31.5) | 0.70 | |
| S-MPM | 4 (4-6) | 6 (4-6) | <0.01 | |
| Hypertension n(%) | 114 (47) | 56 (72) | <0.01 | |
| Ischemic heart disease n(%) | 17 (7) | 12 (15) | 0.04 | |
| COPD/asthma n(%) | 37 (15) | 22 (28) | 0.01 | |
| Diabetes mellitus n(%) | 31 (13) | 18 (23) | 0.03 | |
| Stroke n(%) | 10 (4) | 11 (14) | <0.01 | |
| **Surgery** | | | | |
| Surgery time (min) | 135 (85-180) | 180 (145-245) | | <0.01 |
| Toracotomy No (%) | 123 (50) | 50 (64) | | 0.04 |
| Lobectomy No (%) | 106 (43) | 53 (68) | | <0.01 |
| Bilobectomy No (%) | 6 (15) | 3 (4) | | 0.69 |
| Pneumonectomy No (%) | 5 (2) | 6 (8) | | 0.03 |
| Wedge resection No (%) | 128 (52) | 16 (21) | | <0.01 |
| **Postoperative Outcome** | | | | |
| Hospital LOS (days) | 6 (5-8) | 11 (8-16) | | <0.01 |
| ICU LOS (days) | 3 (2-4) | 6 (4-10) | | <0.01 |
| Chest drainage (days) | 4 (3-6) | 7 (6-9) | | <0.01 |
| ICU readmission No (%) | 1 (0.4) | 10 (13) | | <0.01 |
| 30-day mortality No (%) | 2 (1) | 5 (6) | | 0.01 |
| **Pulmonary Function Tests** | | | | |
| FEV_1_ (%predicted) | 94 ± 18 | 86 ± 20 | | <0.01 |
| FVC (%predicted) | 95 ± 16 | 90 ± 18 | | 0.01 |
| FEV_1_/FVC (%) | 82 (76-87) | 78 (69-85) | | <0.01 |
| DL_CO_ (%predicted) | 84 ± 21 | 76 ± 25 | | <0.01 |
| **Rest Arterial Blood Gases** | | | | |
| PaO_2_ (mmHg) | 79 ± 9 | 75 ± 8 | | <0.01 |
| PaCO_2_ (mmHg) | 36 (33-38) | 35 (32-37) | | 0.06 |
| pH | 7.45 (7.43-7.46) | 7.44 (7.43-7.46) | | 0.46 |
| **Peak Exercise Arterial Blood Gases** | | | | |
| PaO_2_ (mmHg) | 86 (79-92) | 85 (77-91) | | 0.19 |
| PaCO_2_ (mmHg) | 36 ± 5 | 35 ± 4 | | 0.13 |
| pH | 7.37 (7.33-7.39) | 7.36 (7.34-7.39) | | 0.47 |
| **Rest Ventilation and Gas Exchange** | | | | |
| VO_2_ (ml/kg/min) | 4.2 (3.5-5.0) | 4.2 (3.2-4.7) | | 0.34 |
| VCO_2_ (ml/min) | 0.25 ± 0.10 | 0.22 ± 0.09 | | 0.01 |
| V_E_ (l/min) | 10 ± 4 | 10 ± 4 | | 0.25 |
| V_T_ (ml) | 0.57 (0.40-0.74) | 0.53 (0.39-0.68) | | 0.42 |
| f_b_ (bpm) | 18 (15-21) | 18 (15-21) | | 0.51 |
| V_D_/V_T_ | 0.29 (0.23-0.34) | 0.30 (0.24-0.34) | | 0.58 |
| P_ET_CO_2_ (mmHg) | 30 (27-32) | 27 (24-30) | | <0.01 |
| **Peak Exercise Ventilation and Gas Exchange** | | | | |
| VO_2_ (ml/kg/min) | 19.9 (16.5-25) | 18.3 (15.2-22.6) | | 0.01 |
| VCO_2_ (ml/min) | 1.67 (1.30-2.10) | 1.42 (1.10-1.82) | | <0.01 |
| RER | 1.05 (0.92-1.14) | 0.97 (0.87-1.12) | | 0.01 |
| V_E_ (l/min) | 54 (44-68) | 53 (42-65) | | 0.13 |
| V_T_ (ml) | 1.75 (1.37-2.11) | 1.58 (1.13-2.02) | | 0.02 |
| f_b_ (bpm) | 32 (28-36) | 34 (30-38) | | 0.01 |
| V_D_/V_T_ | 0.21 ± 0.07 | 0.22 ± 0.06 | | 0.13 |
| P_ET_CO_2_ (mmHg) | 36 ± 5 | 32 ± 6 | | <0.01 |
| V_E_/VCO_2_ slope | 29 (25-33) | 34 (30-38) | | <0.01 |

BMI=body mass index; COPD=Chronic obstructive pulmonary disease; DL_CO_=diffusing lung capacity for carbon monoxide; f_b_=breathing frequency; FEV_1_=forced expiratory volume in one second; FVC=forced vital capacity; ICU=intensive care unit; LOS=length of stay; PaCO_2_=arterial partial pressure of carbon dioxide; PaO_2_=arterial partial pressure of oxygen; P_ET_CO_2_=partial pressure of end-tidal carbon dioxide; RER=respiratory exchange ratio; S-MPM=Surgical Mortality Probability Model; VCO_2_=carbon dioxide output; V_D_=dead space volume; V_E_=minute ventilation; V_E_/VCO_2_=ventilatory efficiency; VO_2_=oxygen consumption; V_T_=tidal volume
